# Science fiction or clinical reality: a review of the applications of artificial intelligence along the continuum of trauma care

**DOI:** 10.1186/s13017-022-00469-1

**Published:** 2023-03-06

**Authors:** Olivia F. Hunter, Frances Perry, Mina Salehi, Hubert Bandurski, Alan Hubbard, Chad G. Ball, S. Morad Hameed

**Affiliations:** 1 Department of Surgery, University of British Columbia, Vancouver, Canada; 2 T6 Health Systems, Boston, USA; 3 University of California, Berkeley School of Public Health, Berkeley, USA; 4 Department of Surgery, University of Calgary, Calgary, Canada

**Keywords:** Trauma, Artificial intelligence, Machine learning

## Abstract

Artificial intelligence (AI) and machine learning describe a broad range of algorithm types that can be trained based on datasets to make predictions. The increasing sophistication of AI has created new opportunities to apply these algorithms within within trauma care. Our paper overviews the current uses of AI along the continuum of trauma care, including injury prediction, triage, emergency department volume, assessment, and outcomes. Starting at the point of injury, algorithms are being used to predict severity of motor vehicle crashes, which can help inform emergency responses. Once on the scene, AI can be used to help emergency services triage patients remotely in order to inform transfer location and urgency. For the receiving hospital, these tools can be used to predict trauma volumes in the emergency department to help allocate appropriate staffing. After patient arrival to hospital, these algorithms not only can help to predict injury severity, which can inform decision-making, but also predict patient outcomes to help trauma teams anticipate patient trajectory. Overall, these tools have the capability to transform trauma care. AI is still nascent within the trauma surgery sphere, but this body of the literature shows that this technology has vast potential. AI-based predictive tools in trauma need to be explored further through prospective trials and clinical validation of algorithms.

## Background

**Fig. 1:**
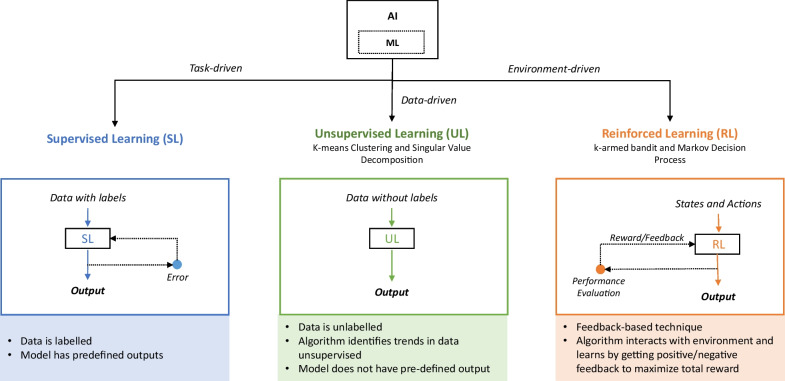
Overview of major types of machine learning. Overview of different types of machine learning (ML): ML is shown as a subset of artificial intelligence (AI). Within ML, there are three subtypes: supervised learning, unsupervised learning, and reinforced learning. Supervised learning is task-driven and uses labeled data to predict a predefined outcome. Unsupervised learning is data-driven and is used to find trends/outputs in unlabeled data. Reinforced learning is environment-driven and uses interaction with the environment to learn

The term artificial intelligence (AI) was first conceived in 1955 by John McCarthy as “the science and engineering of making intelligent machines” [1]. More colloquially, AI can be thought of as a broad term describing an algorithm that performs tasks that would normally require human intervention. Machine learning (ML) is a subtype of AI whereby these algorithms can improve their performance over time with additional experience [2]. There are many ways to classify ML algorithms, but one of the most popular ways is to group them into three main categories: supervised, unsupervised, and reinforced learning (Fig. 1). Supervised learning uses labeled inputs to produce a defined set of outputs of discrete values [2]. Examples of supervised learning includes decision trees, support vector machines (SVMs), regressions, and artificial neural networks (ANNs). Unsupervised learning creates groups from data whereby elements within each group are like each other but dissimilar to other groups; popular unsupervised algorithms include k-means clustering, singular value decomposition, and DBSCAN [2]. Finally, reinforcement learning is a technique that uses interactions with its environment to learn how to behave through trial and error; these include k-armed bandit, Markov decision processes, and SARSA [2].

**Fig. 2:**
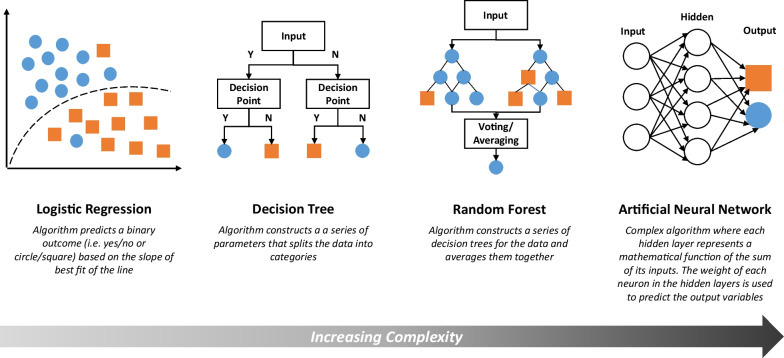
Overview of major types of supervised learning. Continuum of complexity of supervised learning algorithms: While not all types of supervised learning algorithms are shown here, four major illustrative examples—logistic regression, decision tree, random forest, and artificial neural network—are shown along a qualitative continuum from least to most complex. The diagrams are meant to provide a visualization of the algorithm processes whereby the blue circles and the orange squares represent different outcomes

For the purposes of trauma medicine, supervised learning algorithms have been studied the most, and therefore are the focus of this review. Supervised learning algorithms vary significantly in complexity (Fig. 2). More basic supervised ML algorithms are logistic regressions and decision trees [3]. These algorithms are interpretable (and, for decision trees, familiar clinically) but are lower in accuracy due to their relatively low flexibility. Algorithms capable of producing a range of forms include random forests and ANNs. These systems are complex and, while highly accurate, are less transparent to the user. Random forests are effectively an average of many permutations of decision trees made from a data set. ANNs emulate the connections within a brain, with inputs “synapsing” with multiple hidden layers (“interneurons”) via complex equations to deliver predictions [4]. Using AI in trauma requires balancing a model’s sophistication and complexity with transparency and usability.

The purpose of this review is to critically appraise and highlight the different applications of AI/ML that have been studied in trauma care in order to  provide clinicians, hospital administrators, and other non-computer scientist/non-technical audiences a basic understanding of AI/ML, the capabilities of these algorithms, and the potential ways that these may transform trauma care in the future. As injuries cause the greatest reduction in active life years globally and are the leading cause of death in people under 40 [5], ML has the potential to impact global public health through the optimization of processes and improvement of outcomes. With large-scale electronic health record implementation, an unprecedented volume of trauma data are available to train and validate new ML systems [6]. As such, trauma care is primed for AI-based transformation. This article follows the trauma patient’s journey—starting from the point of injury, through triage and arrival at the emergency department, to treatment and outcome prediction—to outline the utility of AI along the entire continuum of trauma care (Table 1) [7–53].

**Table 1:** Studies utilizing artificial intelligence in trauma surgery

Study	Objective	Algorithm type(s)	Input variables	Prediction output(s)	Results (testing set)
*Injury*					
Abdel-Aty et al. [7]	Predict driver injury severity after crash has occurred	MLP Fuzzy adaptive resonance theory	Alcohol involvement Area type Demographics (age, gender) Lighting Peak period Point of impact Seatbelt Speed ratio Trafficway character Vehicle type Weather	Disabling injury/fatality Evident injury Possible injury No injury	73.5% accuracy for the MLP 70.6% accuracy for fuzzy adaptive resonance theory
Al Mamlook et al. [8]	Predict severity of traffic accidents and contributing factors to Traffic Accident Severity	AdaBoost LR Naive Bayes RF	Alcohol or drug involvement Car manufacturing year Clear weather Demographics (age, gender) Driver hazard action Lighting Seatbelt Traffic volume	Fatal or severe crash Minor, possible, or property damage crashes	75.5% accuracy for RF 74.5% accuracy for AdaBoost and LR 73.1% accuracy for Naïve Bayes
Amiri et al. [9]	Predict severity of crashes where driver is > = 65-years-old and hits a fixed object	ANN Hybrid Intelligent Genetic Algorithm	Average annual daily traffic Cause of collision Demographics (age, gender) Facility access Left shoulder Lighting Median type Number of lanes Number of vehicles Right shoulder Road surface condition Surface type Time	Fatal Severe injury Visible injury Complaint of pain Property damage only	0–94.6% accuracy for ANN depending on level of damage predicted 0–78.6% accuracy for hybrid model depending on level of damage predicted Both models best predicted property damage only
Assi et al. [10]	Predict crash injury severity using attributes that can be quickly identified on crash sites	Feed-forward NN SVM Fuzzy C-means clustering based NN Fuzzy C-means based SVM	Area type Day of the week Junction control Junction type Lighting Number of casualties Number of vehicles involved Road class Road surface condition Road type Speed limit Vehicle type Weather	Severe crash Non-severe crash	~ 74% accuracy for SVM-FCM ~ 73% accuracy for SVM ~ 71% accuracy for FNN-FCM ~ 69% accuracy for FNN
Assi et al. [11]	Predict severity of traffic crashes using attributes that can be quickly identified on crash sites	Deep neural network SVM	Age, vehicle Crash type Day Gender, driver Geometry of roadway Lighting Number of persons involved Number of vehicles involved Roadway median separation Speed limit Surface condition Surface type Traffic control Type of vehicle Weather condition	Severe crash Non-severe crash	95% accuracy for DNN ~ 80% accuracy for SVM
Bao et al. [12]	Predict short-term crash risk at weekly, daily, and hourly levels	Spatiotemporal Convolutional Long Short-Term Memory Network	Arterial percentage Commercial area Crash risk Daily vehicle kilometers traveled Freeway percentage Intersections Local road percentage Population Precipitation Pressure Residential area Road density Snowfall Taxi trips Temperature Wind speed	Crash No crash	88.78–99.21% accuracy on weekly level depending on level of spatial resolution used 75.35–96.46% accuracy on daily level depending on level of spatial resolution used 71.02–93.72% accuracy on hourly level depending on level of spatial resolution used
Delen et al. [13]	Predict motor vehicle crash severity and factors that increase risk of severity during crashes	MLP	Age, vehicle Alcohol or drug involvement Demographics (age, sex) Highway Impact location Lighting Role in accident (striking vs struck) Rollover Seatbelt Surface conditions Vehicle orientation in collision Vehicle type Weekend evenings	Fatality Incapacitating injury Minor non-incapacitating injury Possible injury No injury	70.11–89.34% overall accuracy depending on which of the 5 outcome measures were being predicted
Elamrani Abou Elassad et al. [14]	Design a real-time crash prediction model	SVM MLP	Brake Drift angle Lateral gravity Longitudinal gravity RPM Speed Spin angle Throttle Vertical gravity Weather season Yaw angle	Crash occurrence Crash non-occurrence	93.34% average accuracy for MLP 92.00% average accuracy for SVM
Iranitalab et al. [15]	Compare the ability of 4 algorithms to predict traffic crash severity	Multinomial logit Nearest neighbor classification SVM RF	Accident in traffic Alcohol involvement Animal in roadway Debris Demographics (Driver < 25, Driver 13–19, Female, Male) Double bottom trailer Farm equipment Glare Intersection involved Lighting Median type Non-highway work Number of lanes Obstruction in roadway Population group Public/private property Road characteristics Road classification Road surface condition Road surface type Rut, holes, bumps School bus Shoulders Total trucks/buses Traffic control device inoperative, missing, etc. Vehicles Vision obstruction Weather condition Work zone Worn, travel-polished surface	Disabling/fatal injury Visible injury Possible injury Property damage only	High variability by output level predicted; prediction most accurate for “property damage only” and decreased with increasing severity ~ 0–99% prediction accuracy for MNL ~ 5–80% accuracy for NNC ~ 1–95% accuracy for SVM ~ 5–90% accuracy for RF
Mansoor et al. [16]	Predict crash severity based on easily available crash features	kNN DT AdaBoost SVM Feed-forward ANN	Area type Day of the week Intersection control Intersection type Lighting Number of vehicles involved Road class Road surface condition Road type Speed limit Vehicle type Weather condition	Severe crash Non-severe crash	67.1% accuracy for kNN 69.2% accuracy for DTs 71.4% accuracy for AdaBoost 69.7% accuracy for ANN 68.8% accuracy for SVM 76.7% accuracy for a two-layer ensemble model
Taamneh et al. [17]	Predict severity of road traffic injuries in real time	MLP	Accident Type Causality status Day Demographics (age, gender, nationality) Lighting Number of lanes Reason Road surface Seatbelt Speed limit Time Weather Year	Death Severe accident Moderate accidents Minor accidents	65.1% accuracy
*Pre-hospital triage*					
DiRusso et al. [18]	Predict survival of trauma patients based on pre-hospital and emergency room admission data	Feed-forward ANN	Certification level of responder Demographics (age, race, sex) GCS Hct ICD-9-CM E-code Intubation status ISS Time to ED Vitals (sBP, HR, RR, temp)	Survival	0.910–0.912 AUC for the ANN
Kang et al. [19]	Predict need for critical care patients in emergency medical services	Deep learning	Chief complaint Demographics (Age, sex) Mental status Time from symptom onset to visit or EMS contact Trauma Vitals, initial	Need for critical care	0.867 AUC for predicting need for critical care
Kim et al. [20]	Triage patients by casualty likelihood for mass casualty incidents	LR RF NN	Age Consciousness score Vitals (sBP, HR, RR)	Death Survival	0.71–0.88 AUC for LR depending on combination of input variables 0.89 AUC NN 0.87 AUC RF
Liu et al. [21]	Predict injury severity in real time as defined by the need for life-saving intervention in the pre-hospital or emergency department settings	Hybrid system: Basic detection rules + MLP	Pulse pressure Shock index Vitals (SpO2, dBP, sBP, HR, RR, MAP)	Life-saving intervention	69.5–89.8% accuracy depending on defined true positive rate
Nederpelt et al. [22]	Design algorithm to support in-field triage decisions after gunshot wound	Information-aware Dirichlet deep neural network	Alcohol involvement BMI Comorbidity Demographics (age, ethnicity, race, sex) Drug screen GCS GSW anatomical site Time from dispatch to evaluation Transfer status Vitals (sBP, HR, SpO2, RR, temp)	Early massive transfusion Need for major hemorrhage control procedures Shock	0.88–0.89 AUROC for shock depending on input variables used 0.86 AUROC for massive transfusion regardless of input variables used 0.80–0.82 AUROC for hemorrhage control depending on input variables used
*Emergency department*					
Dennis et al. [23]	Predict trauma admission volume, penetrating trauma admissions, and mean ISS	Feed-forward ANN	Center Daily high temperature Daily low temperature Day of week Day of year Precipitation Snow	Mean ISS score Number of penetrating traumas Number of traumas	R = 0.8732 correlation coefficient for all variables
Menke et al. [24]	Predict emergency department patient volumes on a daily level	ANN	Air quality Days of the week Special events Weather	ED volume	95% accuracy of volume within 20 visits of the true volume
Rauch et al. [25]	Predict hourly emergency department volume based on traffic data	Seasonal autoregressive cross-validated models	Historical traffic data (direction and number of vehicles)	ED occupancy	3.21–4.23 patient root-mean square error depending on prediction horizon 2.32–3.25 patient mean average error depending on prediction horizon
Stonko et al. [26]	Predict the volume and acuity of trauma volume in an emergency department	ANN	Daily high Date Day of week ED discharge disposition Injury type ISS Mechanism of injury Precipitation	Mean ISS score per day Number of OR cases per day Number of penetrating traumas per day Number of traumas per day	0.8940 correlation coefficient for all outcome variables
*Workup*					
Batchinsky et al. [27]	Predict need for life-saving interventions based on EKG data	ANN	EKG findings	Life-saving intervention needed No life-saving intervention needed	~ 0.86 AUC
Bektas et al. [28]	Detect craniocervical junction injuries based on CT and patient/injury characteristics	LR ANN	Alcohol intoxication Demographics (age, sex) Falls GCS Motor vehicle accident Motorbike accident Pathology on head CT Pedestrian struck RTS Vitals (HR, MAP, RR)	Presence of craniocervical junction injury	0.794 AUC for LR 0.912 AUC for ANN
Bertsimas et al. [29]	Predict cervical spine injuries in patients < 3 to avoid imaging	OCT GB trees LR	Demographics (age, gender) GCS Mechanism of injury	Presence of cervical spine injury Absence of cervical spine injury	90.43% AUC for OCT 96.69% AUC for GB trees 94.06% AUC for LR
Cheng et al. [30]	Determine accuracy of AI-assisted free fluid detection in Morison’s pouch during FAST examination	Deep learning	Abdominal US	Negative/non-qualified view Negative/qualified view Positive/non-qualified view Positive/qualified view	96.7% accuracy for detecting ascites 94.1% accuracy in classifying qualified and non-qualified images
Dreizin et al. [31]	Use deep learning to segment the volume of pelvic hematomas	Recurrent Saliency Transformation Network	Chest, pelvis, abdominal CT scans	Pelvic hematoma volume	0.81 AUC for predicted volumes (as compared to 0.80 as manually done by radiologists)
Liu et al. [32]	Predict the need for life-saving interventions in trauma patients	LR MLP	GCS HR complexity HR variability Pulse pressure Shock index Vitals (dBP, sBP, HR, SpO2)	Life-saving intervention	0.73–0.94 AUC for LR depending on variables included 0.99 AUC for MLP
Paydar et al. [33]	Predict injury severity from clinical and paraclinical data on blunt trauma injury	SVM KNN Bagging AdaBoost NN	67 features including vital signs, injury organs, and ISS (exact features not listed)	Critically ill Not critically ill	~ 99.24% accuracy for SVM 63.84% accuracy for KNN 99.67% accuracy for Bagging ~ 75.81% accuracy for AdaBoost 51.60% accuracy for NN
*Intervention and outcome*					
Abujaber et al. [34]	Predict risk of prolonged mechanical ventilation with TBI	LR ANN SVM RF C.5 DT	AIS per body region Blood transfusions CT scan findings Date/time of injury Demographics (age, gender, race) GCS In-hospital complications Intubation status ISS Comorbidities Mechanism of injury Outcome and date of disposition Performed procedures Time of ED admission Vitals, on ED arrival	Prolonged mechanical ventilation (> 7, > 10, or > 14 days) No prolonged mechanical ventilation	73–75% accuracy for LR 69–77% accuracy for ANN 74–79% accuracy for SVM 71–75% for RF 66–71% for C.5 DT
Ahmed et al. [35]	Predict mortality in patients admitted to trauma surgery ICU	Deep-FLAIM Gaussian Naïve Bayes DT KNN Linear Discriminant Analysis	Acute Physiology Score III Angus Criteria of Sepsis Laboratories (albumin, anion gap, BUN, creatinine, glucose, INR, lactate, platelets, PT, PTT, serum electrolytes) Logistic Organ Dysfunction System Oxford Acute Severity of Illness Score qSOFA SAPS SAPS II Sepsis diagnosis using Martin Sepsis et al SIRS SOFA	Survival	92.25% accuracy for Deep-FLAIM 80.07% accuracy for GNB 89.59% accuracy for DT 84.94% accuracy for KNN 81.84% accuracy for LDA
Becalick et al. [36]	Compare ability of ANN to predict outcome after injury with UK TRISS	ANN	AIS for each body region Demographics (age, gender) GCS Injury type Vitals (sBP, HR, SpO2, RR)	Survival	89.6% accuracy for both ANN and UK TRISS; however, accuracy higher for ANN for predicting survival while UK TRISS better predicts death
Christie et al. [37]	Predict dynamic/up-to-date risk of complications and identify patient-specific modifiable factors to adjust patient trajectory after severe injury	Ensemble machine learning algorithm	Alcohol or drug involvement APACHE-II Coagulation marker Demographics Denver Post-Injury Multiple Organ Failure Score Fluid, colloid, blood, and medication administration GCS Inflammation markers Injury characteristics Input/output data ISS Past medical history Ventilator parameters Vitals	Acute Respiratory Distress Syndrome Blood transfusion Coagulopathy/coagulopathic trajectory Length of Mortality Organ failure Venous thromboembolic events	0.76–0.98 AUC for predicting death depending on time since admission 0.87–0.96 AUC for multi-organ failure 0.82–0.86 AUC for venous thromboembolic events at 96-120 h 0.84–0.88 AUC for transfusion 0.71–0.83 AUC for acute respiratory distress syndrome 0.45–0.74 AUC for coagulopathic trajectory
Demsar et al. [38]	Predict patient outcome after initial damage control surgery	Classification trees Naïve Bayes classifier	Bicarbonate excess in ICU Catecholamine administration Estimated blood loss Physician impression of coagulopathy during operation PT in ICU Type of closing Worst arterial carbon dioxide tension Worst mean blood pressure Worst partial active thromboplastin time Worst pH Worst pH value at ICU Worst sBP	Survival	82.4% accuracy for classification trees 79.4–80.9% accuracy for Naïve Bayes
DiRusso et al. [39]	Compare ability of ANN and LR to predict pediatric trauma death	ANN LR	Demographics (age, sex) GCS Intubation status ISS NISS Pediatric Trauma Score RTS Vitals (sBP, HR, RR)	Survival	0.964 AUROC for LR 0.961–0.966 AUROC for ANN
El Hechi et al. [40]	Predict 30-day outcomes in patients undergoing emergency operations	OCT	Comorbidities Demographics Laboratory values Wound characteristics	30-day morbidity 30-day mortality Occurrence of 18 complications	0.93 c-statistic for predicting mortality 0.83 c-statistic for predicting morbidity
Gorczyca et al. [41]	Compare an algorithm they developed against established risk prediction models (BLISS, HARM, and TMPM)	Stacked generalization of 5 different ML algorithms (LR with Elastic Net Penalty, RF, GB Machine, NNs)	Comorbidities Demographics (age, gender) GCS ICD-9 codes Injury mechanism Injury type Intent of trauma	Risk of death	96.8% accuracy when only ICD-9 codes used as input 97.6% accuracy when all inputs utilized Algorithm equaled or improved as compared to established risk prediction models
Hale et al. [42]	Predict clinically relevant TBI in pediatric patients	ANN	Demographics (age, sex) GCS Injury mechanism Loss of consciousness Radiologist-interpreted CT scan with 17 variables identified Severity of injury mechanism	Clinically relevant TBI defined by needing neurosurgical procedure, intubation > 24 h, hospitalization > 48 h, or death	0.9907 AUC for detecting CRTBI
Ji et al. [43]	Predict final outcome and ICU length of stay for trauma patients	Classification and regression tree C4.5 AdaBoost SVM NN LR	AIS by body part Blunt vs penetrating injury Comorbidities Complications Demographics (age, gender) GCS Intubation ISS Method of injury Provided fluids Role in accident Safety measures used during injury Vitals (BP, HR, RR)	Discharge location ICU length of stay Survival	69.4–72.9% accuracy for LR depending on input variables 70–73% accuracy for AdaBoost depending on input variables 68–75.2% accuracy for C4.5 depending on input variables 75.6–77.6% accuracy for CART depending on input variables 73–79% accuracy for SVM depending on input variables 67.2–79.04% accuracy for NN depending on input variables
Matsuo et al. [44]	Predict morbidity and mortality after TBI using parameters that are quickly and easily available in emergency care	Ridge regression Least absolute shrinkage and selection operator RF GB Extra trees DT Gaussian naïve Bayes Multinomial naïve Bayes SVM	Abnormal pupillary response Age sBP CT findings GCS Laboratories (CRP, fibrin/fibrinogen degradation products, glucose) Major extracranial injury	Death Poor outcome based on Glasgow Outcome Score	71.7% accuracy for Gaussian NB for morbidity 90.2% accuracy for GB for morbidity 91.7% accuracy for RF for morbidity 78.2% accuracy for SVM for morbidity 95.5% accuracy for RF for mortality 88.6% accuracy for ridge regression for mortality 88.5% accuracy for SVM for mortality Not all algorithms had test results shown in the paper
Maurer et al. [45]	Design and validate a smartphone-based risk calculator for trauma patients	OCT	AIS by body region Comorbidities Demographics (age, ethnicity, sex, race) GCS Mechanism of injury Vitals (sBP, HR, SpO2, RR, temp)	Acute kidney injury Acute respiratory distress syndrome Cardiac arrest requiring CPR Deep surgical site infection Deep vein thrombosis In-hospital morality Organ space surgical site injury Overall morbidity Pulmonary embolism Severe sepsis Unplanned intubation	0.941 c-statistic for predicting mortality in penetrating injury 0.884 c-statistic for predicting mortality in blunt injury 0.777 c-statistic for predicting morbidity in penetrating injury 0.753 c-statistic for predicting morbidity in blunt injury 0.689–0.835 c-statistics for predicting individual complications
Nourelahi et al. [46]	Predict “favorable” or “unfavorable” outcome after 6 months in severe TBI	LR RF SVM	Demographics (age, sex) GCS motor response Laboratories (glucose, PT-INR) Pupil reactivity Rotterdam index	GOSE = < 4 (“Unfavorable”) GOSE > 4 (“Favorable”)	78% accuracy for all three model types
Pang et al. [47]	Predict outcomes of severe TBI patients	LR NN DT Bayesian network Discriminant analysis	Coagulopathy Demographics (age, ethnicity, gender) Mechanism of injury Pre- and post-resuscitation GCS Pre- and post-resuscitation pupillary anomaly Traumatic subarachnoid hemorrhage Vitals (hypotension, hypoxia)	Glasgow Outcome Scale	73.1% overall accuracy for DTs 70.51% overall accuracy for LR 66.39% overall accuracy for discriminant analysis 65.67% overall accuracy for Bayesian network 63.38% overall accuracy for NN
Rashidi et al. [48]	Determine if a burn-trained algorithm could be generalized to a non-burned trauma surgery population to predict acute kidney injury	LR KNN RF SVM MLP	Central venous pressure Demographics MAP Laboratories (creatinine, NGAL, NT-proBNP) Urine output	Acute kidney injury	~ 70–75% accuracy for all algorithms when all input variables included Variation of all algorithms ~ 20–90% depending on which inputs included
Rau et al. [49]	Predict survival probability of trauma patients by the addition of a large number of input variables	LR SVM NN	AIS in different body regions Comorbidities Demographics (age, sex) GCS ISS Laboratory results (WBC, RBC, Hgb, Hct, platelets, neutrophils, INR, glucose, Na + , K + , BUN, Creatinine, aspartate, AST, ALT) RTS TRISS Vitals (dBP, sBP, HR, RR, temp)	Survival	97.9% accuracy for LR 98.0% accuracy for SVM 98.3% accuracy for NN
Schetinin et al. [50]	Predict trauma severity in trauma surgery patients	Bayesian averaging over DTs	Demographics (age, gender) GCS Injury severity (head, face, neck, thorax, abdomen, spine, upper extremity, lower extremity, and external) Injury type Vitals (BP, RR)	Death	87.5–98.7% accuracy depending on the number of injuries present
Shahi et al. [51]	Predict outcomes in pediatric patients with blunt solid organ injury	Deep Learning	Blood transfusion Clinical events (e.g., intubation, CPR) CT grade of injury Demographics (age, gender) ED TEG values FAST exam findings Fluid administered GCS Laboratory values (Hgb, INR, base deficit, lactate) Multiple solid organ injuries Presence of head injury SIPA scores Weight Vitals (BP, HR)	Mortality Failure of non-operative management Massive transfusion Successful non-operative management without intervention	90.0–90.5% accuracy for massive transfusion depending on prediction horizon 82.4–83.8% accuracy for failure of non-operative management depending on prediction horizon 91.9% accuracy for mortality across prediction horizons 86.9–90.3% accuracy for successful non-operative management without intervention Note: only validation data shown
Staziaki et al. [52]	Predict extended length of stay and ICU admission in trauma of the torso	SVM ANN	AAST grading CT imaging findings Demographics (age, sex) GCS Laboratories (Hct, Hgb, lactate) RTS Vitals	Extended length of stay ICU admission	77–82% accuracy for SVM for ICU admission depending on inputs used 77–83% accuracy for ANN for ICU admission depending on inputs used 58–73% accuracy for SVM for extended length of stay depending on inputs used 65–77% accuracy for ANN for length of stay depending on inputs used
Tsiklidis et al. [53]	Predict trauma patient survival and identify patient warning signs	GB	Demographics (age, gender) GCS Vitals (sBP, HR, SpO2, RR, temp)	Deceased Survived	0.924 AUC for predicting death

AAST = American Association for the Surgery of Trauma; AIS = Abbreviated Injury Scale; ALT = alanine transaminase; ANN = artificial neural network; AST = aspartate aminotransferase; AUC = area under the curve; AUROC = area under the receiver operating characteristic; BUN = blood urea nitrogen; CRP = c-reactive protein; dBP = diastolic blood pressure; DT = decision tree; GB = gradient booster; GCS = Glasgow Coma Scale; GOSE = Glasgow Outcome Scale Extended; Hct = hematocrit; Hgb = hemoglobin; HR = heart rate; INR = international normalized ratio; ISS = Injury Severity Score; kNN = k-nearest neighbor; LR = logistic regression; MAP = mean arterial pressure; MLP = multilayer perceptron; NGAL = biomarker for acute kidney injury; NISS = New Injury Severity Score; NN = neural network; OCT = optimal classification trees; PT = prothrombin time; PTT = partial thromboplastin time; qSOFA = Quick SOFA; RBC = red blood cell count; RF = random forest; RR = respiratory rate; RTS = Revised Trauma Score; SAPS = Simplified Acute Physiology Score; SAPS II = Simplified Acute Physiology Score II; sBP = systolic blood pressure; SIPA = Shock Index, Pediatric Age-adjusted; SIRS = systemic inflammation response syndrome; SOFA = Sequential Organ Failure Assessment; SpO2 = oxygen saturation; SVM = support vector machine; temp = body temperature; TRISS = Trauma Injury Severity Score; and WBC = white blood cell count

## Injury prediction

AI applications in trauma begin before injury. While trauma and emergency physicians use heuristics and patterns to predict when injuries are most likely to happen (e.g., date, time of day, weather [54]), these approaches lack sensitivity and adaptability. AI can help refine injury prediction.

Within injury prediction, motor vehicle crashes (MVCs) are the most studied. MVC studies can be further subdivided into crash occurrence and crash severity prediction studies. Crash occurrence models are complex and appear to be earlier in development. MVC occurrence prediction has been modeled using simulators that predict crash occurrence or non-occurrence based on car movement (e.g., gravity, drift angle) and environment (e.g., weather) [14]. Elamrani Abou Assad et al. were able to achieve prediction accuracies of 92.00% for an SVM and 93.34% for a multilayer perceptron (MLP), which is a type of ANN [14]. While these accuracy levels are very high, simulator studies have limited utility in healthcare as every road, car, and driver cannot be tracked for up-to-date predictions, and limited research has been done to apply this principle to populations due to the spatial and temporal complexities of this process. Bao et al. [12] is one of the few who has approached this task by using a spatiotemporal convolutional long short-term memory network to predict short-term crash risk at a weekly, daily, and hourly level within Manhattan using historical crashes, taxi GPS, road networks, land use, weather, and population. This model achieved a 71.02–99.21% prediction accuracy based on the spatial and temporal resolution used (with increasing accuracy at lower resolutions); however, tools like this may be impractical for prospective use due to the processing power and volume of data required [12].

Crash severity studies are more established and predict injury acuity from easily identifiable crash scene characteristics [7–11, 13, 15–17]. Inputs often include road (e.g., speed limit, surface type), vehicle/driver (e.g., driver/vehicle age, vehicle type), and environmental characteristics (e.g., weather, time) [7–11, 13, 15–17]. These algorithms are trained to predict injury level based on predefined categories; these are usually either basic, such as severe/not severe, or more complex, such as no injury/possible injury/non-incapacitating injury/incapacitating injury/fatality [7–11, 13, 15–17]. The vast majority of studies have focused on different types of ANNs, such as MLPs or deep neural networks due to their capability to handle highly complex data inputs, and have found AI can predict severity with an accuracy of 0–96% depending on the study [7–11, 13, 15–17]. This enormous variability is due to several factors, including differences in data input (such as geography and quantity/quality of crash data) but also the output type. Of the MVC crash severity studies examined, those that tried to predict a greater number of output categories tended to decrease their accuracy [7–11, 13, 15–17], as there was fewer representative data per category to train the algorithm. These studies suggest that MVC severity prediction could help prepare first responders and hospitals on likelihood of injury severity if the appropriate input, output, and algorithm type is selected. Other mechanisms of injury, such as interpersonal violence and self-harm are less developed in current injury prediction research.

Building and validating these predictive algorithms has clinical and public health applications. Clinically, an AI-based application could be used by 911 dispatchers based on caller information to predict injury severity and more accurately inform EMS prioritization and response. Systemically, understanding the variables associated with injury severity can support harm-reduction and injury prevention public health strategies [12, 14, 55].

## Pre-hospital triage

Once injury occurs, AI can help triage patients before hospital arrival. Currently, remote triage takes time and relies on (1) EMS to contact hospitals when high-acuity patients are *en route* and (2) effective communication between the EMS team and the receiving physician. AI has been shown to predict the need for critical care/life-saving interventions to help stratify incoming trauma patients pre-hospital both generally [17, 19–21] and in specific trauma subtypes, such as gunshot wounds and after resuscitation [22, 33]. The ability to predict the need for life-saving interventions can help inform hospital selection, allowing EMS to route to hospitals with the capacity to handle the necessary care for their patient. This could be especially useful in rural and remote settings where decisions must be made about air evacuation. Further, more detailed information and predictions about patients *en route* to hospital could help receiving centers prepare for the upcoming trauma activation, such as through allocating appropriate resource/operating rooms or ensuring available staff.

Algorithm inputs range in complexity from 6–8 inputs mostly comprising vitals, such as in the case of Liu et al. and Kim et al. [20, 21], to more complex analyses that consider time to dispatch, basic laboratories, and injury characteristics [18, 19, 22]. Almost all the studies that were examined for this paper employed types of ANNs to elucidate this relationship, and studies that used a greater number of variables often (but not always) had greater accuracy (AUC 0.82–0.912) as compared to those with fewer input variables (AUC 0.71–0.88) [18–22].

Remote triage systems may be efficient if they require minimal data input by the EMS team and can be used to ensure appropriate resources available for patients on arrival to the hospital. However, remote triage applicability may be limited as there is a trade-off between increased accuracy and necessary data volume. There may be a threshold where the inconvenience to EMS of managing high data volume surpasses the relative accuracy increase in triage. Kim et al. [20] used data that could be collected on wearable devices—including systolic blood pressure, heart rate, respiratory rate, and a modified consciousness score—in addition to patient age to predict patient likelihood of death. Using an ANN, they were able to achieve an AUC of 0.89 with this method, showing highly accurate prediction with minimal human intervention [20]. The clinical meaning of a difference between an AUC of 0.89 based on this minimal-input wearable and 0.912 based on an intensive high-input algorithm would need to be elucidated in future study; however, the use of wearable sensors appears promising and may allow for dynamic prediction.

## Emergency department volumes

AI has been shown to predict trauma volumes within the emergency department (ED) [23–26]. Inputs reflecting human activity and environmental conditions such as date, traffic, special events, precipitation, temperature, and air quality, are the basis of these algorithms have been used in previous studies [23–26]. Like algorithms predicting crash occurrence in MVCs, predicting ED volumes relies on ANNs to capture the complexity of the relationship between large-scale patterns of human behavior and individual center-level outputs. Further, unlike in previously discussed algorithms in this paper that use AI to predict categorical variables, these algorithms are tasked with continuous outputs.

Unfortunately, differences in data reporting/statistical analysis in the current literature makes cross-comparison between studies difficult. Stonko et al. [26] and Dennis et al. [23] used correlation coefficients to show that ANNs could be used to predict mean ISS, total number of traumas, and number of penetrating traumas in a given day with a correlation of 0.87–0.89. Menke et al. [24] and Rauch et al. [25] used deviations from the true value/average error to show efficacy, showing the predicted ED volume falling within 20 visits of the true volume 95% of the time and a mean average error of 2.32–3.25 patients, respectively. Overall, these systems show lower accuracy at extreme ends of the spectrum (very low- and high-volume days). Each study requires more robust statistical analysis to show accuracy, and thus, the ability to draw conclusions about the future applications in trauma is limited; however, based on information available, they appear to be able to predict volume and acuity on average, which has important implications for trauma care optimization.

Better prediction of trauma volumes can not only improve resourcing for cost savings to the healthcare system but can also lead to better patient outcomes when there is appropriate capacity available to treat each case. However, more investigation will be needed about the adaptability of these algorithms to shifting patterns in human behavior in the wake of the COVID-19 pandemic.

## Initial assessment

Once a patient arrives at the hospital, AI can support initial diagnostic and therapeutic decision-making through patient severity assessments. Predicting patient severity at presentation is a broad category of algorithms that includes prediction of prognosis and decision support.

### Predictive analytics

Prediction of prognosis can help differentiate patients who are critically ill versus those who are not and identify those who will need life-saving interventions. In practice, an AI-based tool in this setting would use easily assessed variables (e.g., vitals, GCS) inputted by the receiving trauma team to initially determine patient prognosis and need for interventions. Studies have taken dramatically different approaches to addressing this task. Liu et al. [32] developed an MLP that used vitals, demographics, and GCS to determine the need for life-saving interventions at ED presentation with an achieved AUC of 0.99. Although limited by small sample size, the algorithm’s performance is promising for further development. Batchinksy et al. [27] used ECG data alone to determine the need for life-saving intervention with an AUC of 0.86. Importantly, in both cases, these variables are readily available in the ED, making these algorithms amenable to use in high-stakes presentations.

### Diagnostics and clinical decision support

Concomitant with determining need for life-saving intervention, clinicians in the ED often need to investigate and rule out injuries on CT, such as cervical spine injury (CSI). Extensive work has gone into creating guidelines to aid physicians in determining when imaging is needed as part of CSI workup, [56]. Despite evidence-based guidelines, imaging is often inappropriately used, with clinically relevant injury found in just 2% of imaged patients [56]. Bektas et al. [28] compared a logistic regression with an ANN to supplement CT in detecting CSI. The ANN had a significantly better negative predictive value than the logistic model at 97.3% versus 87.9%, respectively [28]. The ANN also had a positive predictive value of 100% and detected 2 CSIs that were missed on CT alone [28]. Furthermore, AI can support decision-making in pediatrics where imaging over-use is of greater concern due to carcinogenic irradiation [29]. Using GCS, age, gender, and injury mechanics, an optimal classification tree algorithm predicted CSI in patients < 3 years old with a 93.38% sensitivity and 82.34% specificity [29]. Other studies have demonstrated that AI can assess pelvic hematoma on CT imaging [31]. Volume of pelvic free fluid is used clinically to predict the need for transfusion and angioembolization yet is challenging and time-consuming to quantify on CT [31]. Dreizin et al. [31] developed AI capable of segmenting these CTs to produce reliable volume measurements; this algorithm had results on par to physician judgment with much less time and effort investment, with AUC of 0.81 as compared to an AUC of 0.80 when manually done by radiologists.

AI can supplement other imaging in trauma workup [30, 31, 52]. Ultrasound, while indispensable in trauma evaluation, is limited by its wide sensitivity range (28–100%) [30]. Cheng et al. [30] designed a model that interprets free fluid in Morison’s pouch during FAST exams after torso trauma. The model was trained to not only to detect free fluid, which it did with 96.7% accuracy, but to also determine if the image captured on ultrasound was qualified to make such predictions, which it could determine with 94.1% accuracy. Further studies have used ML to accelerate workup, improve diagnostic accuracy [31], and reduce unnecessary imaging [28, 29].

AI has a potential role in imaging workup in situations where clinicians must rapidly interpret imaging to inform patient management. In high-acuity settings where time is of the essence, these algorithms could evaluate images faster than and with equal or superior accuracy to human review, allowing for the identification of pathology more rapidly and precisely.

## Outcomes

Trauma patients are a heterogeneous group at high risk of complications, including but not limited to organ failure, cardiac arrest, infection, respiratory distress, shock, stroke, and death [40, 45]. Owing to their heterogenicity and rapidly changing status, it remains challenging for physicians to predict a clinical course for these patients in hospital. Numerous non-AI-based risk prediction tools exist for these complications and outcomes, but these tools lack the ability to intelligently adjust the weight of input variables and instead are linear and additive [40]. As such, it is unsurprising that much of the body of the literature around AI in trauma is centered on intervention and outcome prediction. Within intervention and outcome prediction, there are three main types of algorithms: complication prediction, survival prediction, and discharge prediction.

### Complication prediction

ML has been studied to assess its ability to perform risk prediction and accurate prognostication of clinical outcomes in trauma patients [37, 40, 45, 48]. An ideal AI-based tool for complication prediction would either (1) use variables that are readily available to the trauma team after a brief workup (i.e., vitals, comorbidities, injury factors/TRISS scores ± laboratory results) to help identify which complications a patient is most at risk of and which interventions have the greatest possibility of mitigating these complications or (2) use variables that are collected post-intervention (i.e., using all the same variables above but with invention-related inputs) to identify likely downstream postsurgical complications. While complication prediction would have high clinical utility, this type of prediction is technically challenging due to the many potential complications these patients can encounter. To predict an output, there needs to be sufficient examples of that output in the training dataset to determine the relationship between the inputs and the output. With a perfect dataset and infinite computing power, all outputs could be predicted with equal accuracy; however, in practice, data limitations can result in wildly variable capabilities to predict individual outputs within a single algorithm. Christie et al. [37], for example, looked at 7 complications and was able to predict their occurrence with an AUC 0.45–0.74. Maurer et al. [45] looked at 11 complications and achieved c-statistics of 0.689–0.835. As such, individual algorithms that have been developed may be able to accurately predict certain complications but may not be reliable at predicting the full suite of complications that may befall a patient.

Further, with the ever-changing condition of trauma patients, the ideal risk calculator could dynamically alter predictions in real time and identify modifiable factors to change outcomes [37]. Christie et al. [37] designed the “SuperLearner,” an algorithm that incorporates data across time and re-evaluates mortality and complication risk. Although, SuperLearner’s variability in prediction by complication means it may not yet be ready for clinical application, tools that can dynamically adjust predictions over time would have exciting applications in trauma care [37]. In the future, machine learning merged with causal inference methods may be able to predict which treatment would provide the best outcome and could be the basis of precision medicine in trauma.

### Survival prediction

Within outcome prediction, survival prediction is by far the most studied. As such, there is diversity in algorithm choice, input variables, and prediction accuracies within this space. The most basic of these algorithms use inputs such as comorbidities, demographics, GCS, vitals, and injury data [36, 41, 50, 53]. While studies use several different algorithms, they are able to consistently achieve accuracy levels of > 89% with some as high as 97% [36, 41, 50, 53]. As these algorithms become more complicated, they also incorporate laboratory results, imaging findings, currently available scoring systems (e.g., TRISS), and interventions [35, 38, 39, 44, 49, 51]. However, increasing the input complexity does not always increase accuracy. These models are consistently able to predict at accuracies of > 82% and as high as 98% [35, 38, 39, 44, 49, 51]. The small gains in accuracy (and in some studies, a drop in accuracy) relative to simpler models may be due to overfitting, whereby increasing the number and specificity of input variables creates an algorithm that is perfectly trained to predict based on the training dataset but is unable to generalize to new datasets.

**Fig. 3:**
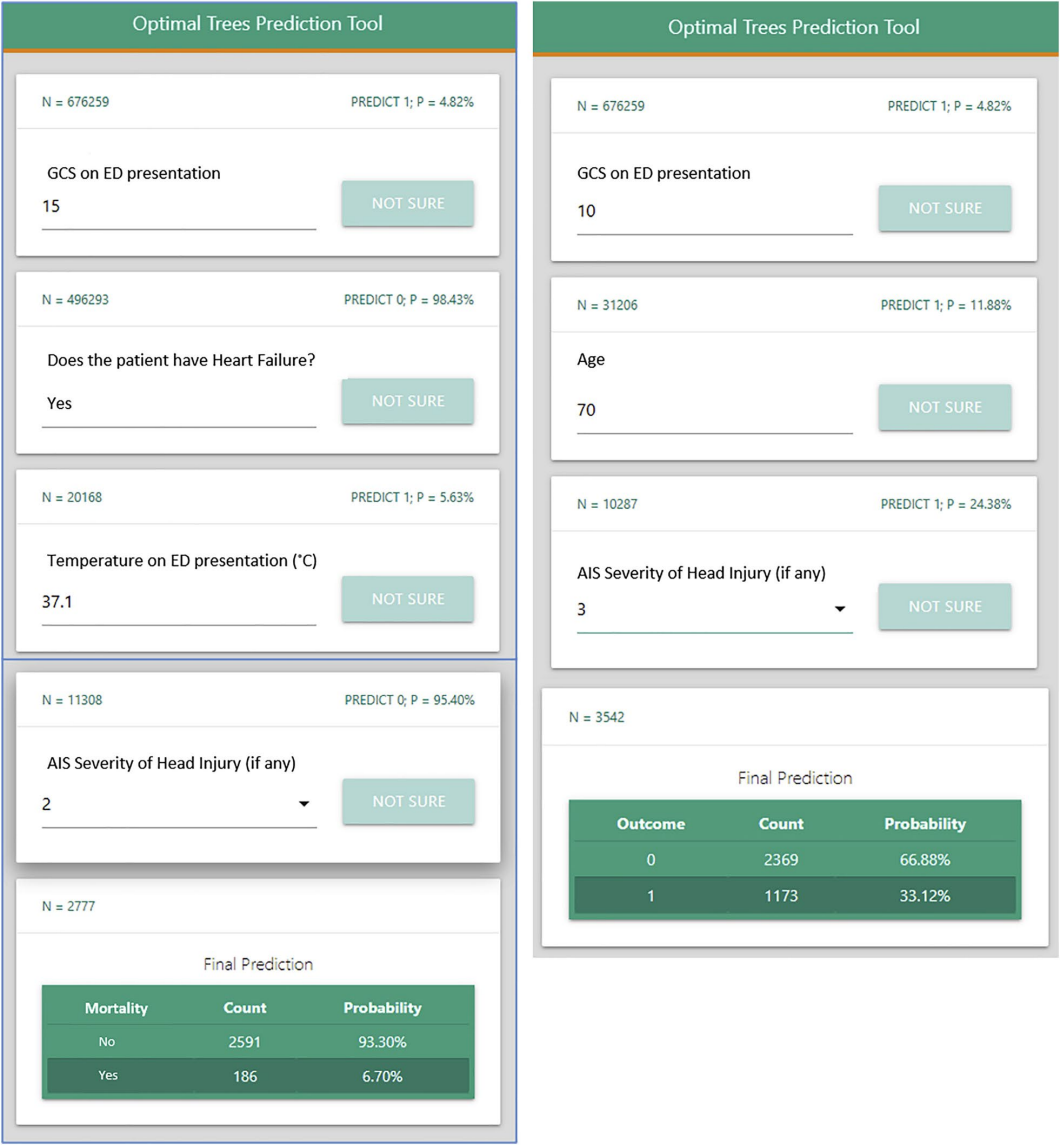
Trauma outcome predictor (TOP) example screen shot. This screenshot of the TOP interface shows how clinicians can input variables based on clinical assessment to predict mortality after blunt injury. The differences between the left and right panels are due to the algorithm’s ability to adjust the questions asked based on answers to previous questions; in this case, the differences in GCS answers prompt the algorithm to diverge in its input variable requirements. Reprinted from Surgery, Vol 171/6, El Hechi M, Gebran A, Bouardi HT, Maurer LR, El Moheb M, Zhou D et al. Validation of the artificial intelligence-based trauma outcomes predictor (TOP) in patients 65 years and older, Page 1689., Copyright (2022) with permission from Elsevier and the original authors

The trauma outcome predictor (TOP) is one such algorithm that has been validated to predict mortality, as well as 9 other complications [45, 57]. It uses data such as demographics, vital signs, mental status, comorbidities, and injury characteristics to feed an optimal classification tree algorithm, which can predict mortality and morbidity with c-statistics up to 0.941 [45]. TOP is an excellent example of what a survival prediction tool would look like in clinical practice, adjusting the necessary input questions based on previous answers in order to predict mortality and morbidity (Fig. 3) [57]. Tools such as TOP could be used by clinicians to assess survival risk in order to plan the intervention and management of patients, as well as inform palliation and end of life discussions.

### Discharge prediction

Understanding discharge disposition and length of stay can help health systems prioritize bed allocation, begin discharge planning in advance, and set realistic expectations for patients and families post-injury. This is especially important post-traumatic brain injury (TBI) where long-term effects can be unpredictable. Pang et al. [47] and Nourelahi et al. [46] compared the efficacy of several algorithm types to predict Glasgow Outcome Scoring for patients’ post-TBI. Both used demographics, GCS, and pupillary responses (with some additional unique inputs per study) and were able to achieve accuracy of 63–78% [46, 47]. Compared to other clinical applications of AI in trauma discussed in this paper, TBI outcome appears to be of lower accuracy, likely due to the high variability in patient recovery post-injury. Length of stay is also a burgeoning area of discharge prediction studies. Staziaki et al. [52] and Ji et al. [43] used several algorithms, including SVM and ANN, to predict duration of hospitalization. Both papers tested a variety of combinations of input variables, including but not limited to demographics, GCS, vitals, and injury scoring, to achieve accuracy levels of 58–79% for SVM and 65–79% for ANN [43, 52].

## Speech interpretation: supporting the trauma care continuum

Narrative clinical documentation in trauma is often difficult to analyze in real time as it is not entered as discrete and time stamped data elements, which is critical for clinically relevant algorithms. Natural language processing (NLP) and automatic speech recognition (ASR) are two fields of AI that can relieve the burden and time of converting speech notes to text and provide higher-quality data input. Research by Blackley et al. [58] found that speech recognition saves time, increases efficiency, and allows for quicker and more relevant documentation. AI related to speech/audio can also help diagnose pneumonia, asthma, and other infections. For example, investigators using cough data [59] achieved 100% asymptomatic COVID-19 detection rate and 88% accuracy on all subjects. Converting narrative data to structured data using NLP/ASR would be potentially transformative in fast-paced and data-rich trauma resuscitation environments, where critical decisions are often made without integration of all available information.

## Conclusions

AI in trauma surgery has numerous applications and proven efficacy. As more studies validate new or existing algorithms, trauma analytics are likely to shift away from rudimentary scoring methods toward more dynamic and accurate AI decision support tools. These tools are applicable from the point of injury through to surgical follow-up.

In order to begin to fulfill the potential of AI, trauma systems must adapt  compatible electronic health records and reporting systems to support real-time data collection and integration. Existing AI systems must be evaluated prospectively to demonstrate replicability as compared to algorithms trained on retrospective data. More ML systems must be able to dynamically adjust their predictions as patient status changes. Algorithms need to be paired with interpretable graphical user interfaces so that they can be used by clinicians and not just computer scientists.

AI has a promising role within trauma surgery practice and is worth the time and investment needed to prove and establish its specific uses. Given the technical expertise required to design, evaluate, and validate these algorithms, this endeavor will require interdisciplinary collaboration between physicians, computer scientists, statisticians, and administrators. These tools have the promise of changing clinical practice and improving patient outcomes and population health.

## Data Availability

Data sharing is not applicable to this article as no datasets were generated or analyzed during the current study.

## Abbreviations

AI: Artificial intelligence
ANN: Artificial neural network
ASR: Automatic speech recognition
AUC: Area under the curve
CT: Computerized tomography
CSI: Cervical spine injury
ECG: Electrocardiogram
ED: Emergency department
EMS: Emergency medical services
GCS: Glasgow Coma Score
ISS: Injury Severity Score
ML: Machine learning
MLP: Multilayer perceptron
MVC: Motor vehicle crash
NLP: Natural language processing
SVM: Support vector machine
TBI: Traumatic brain injury
TRISS: Trauma Injury Severity Score
